# Bisphenol AF Induces Hepatic Steatosis via Succinate–SUCNR1-Mediated Macrophage–Hepatocyte Interactions: An Adverse Outcome Pathway Study in Male C57BL/6 Mice

**DOI:** 10.3390/ijms26199720

**Published:** 2025-10-06

**Authors:** Ning Wang, Jing Leng, Huimin Zhang, Jing Xu, Xiaoqi Yu, Kelei Qian, Zhiqing Zheng, Mengchao Ying, Gonghua Tao, Ping Xiao, Xinyu Hong

**Affiliations:** Shanghai Municipal Center for Disease Control and Prevention, Institute of Chemical Safety Evaluation/Key Laboratory of Environmental and Health Impact Assessment of New Pollutants, 1399 at Shenhong Road, Shanghai 201107, China; wangning@scdc.sh.cn (N.W.); lengjing@scdc.sh.cn (J.L.); zhanghuimin@scdc.sh.cn (H.Z.); xujing@scdc.sh.cn (J.X.); yuxiaoqi@scdc.sh.cn (X.Y.); qiankelei@scdc.sh.cn (K.Q.); zhengzhiqing@scdc.sh.cn (Z.Z.); yingmengchao@scdc.sh.cn (M.Y.); taogonghua@scdc.sh.cn (G.T.); xiaoping@scdc.sh.cn (P.X.)

**Keywords:** Bisphenol AF, SUCNR1, succinate, hepatic steatosis, adverse outcome pathway, macrophage–hepatocyte interaction

## Abstract

Bisphenol AF (BPAF) exposure is increasingly linked to metabolic disorders, yet the molecular initiating events (MIE) and key events (KE) leading to hepatic steatosis remain unclear. We constructed an adverse outcome pathway (AOP) to mechanistically connect BPAF-triggered macrophage–hepatocyte crosstalk to liver fat accumulation. Male C57BL/6 mice received daily oral gavage of 0, 0.5, 4, or 32 mg kg^−1^ BPAF for 90 d, and Transwell co-cultures of RAW264.7 macrophages and AML12 hepatocytes were used for in vitro validation. Targeted metabolomics, western blotting, and lipid staining quantified succinate, pathway proteins, and steatosis. BPAF dose-dependently increased serum succinate (BMD = 6901.95 nM) and hepatic triglyceride (TG) (BMD = 874.26 nM). Cryo-EM docking revealed BPAF binding to SUCNR1 at 2.9 Å, disrupting the inactive-state conformation. In co-culture, BPAF-exposed macrophages released succinate that bound hepatocyte SUCNR1, suppressed Akt phosphorylation, and activated JNK. These KEs led to a 40% increase in lipid droplets and elevated TG, total cholesterol (TC), and free fatty acids (FFA) without liver weight gain. We propose the first AOP for BPAF-induced hepatic steatosis: BPAF–SUCNR1 binding (MIE) → macrophage succinate release (KE1) → SUCNR1-mediated Akt inhibition/JNK activation (KE2–4) → hepatic lipid accumulation (KE5) → steatosis (AO). These findings provide mechanistic insight for chemical risk assessment of BPAF and structurally related bisphenols.

## 1. Introduction

BPAF has been increasingly recognized as a potent metabolic disruptor. Recent toxicological studies have demonstrated that BPAF exposure is associated with enhanced lipogenesis and oxidative stress in hepatocytes [1,2]. Consistent with the Adverse Outcome Pathway (AOP) framework established by the OECD, BPAF may initiate hepatic steatosis via receptor-mediated signaling pathways, particularly through the succinate–SUCNR1 axis [3].

Recent human biomonitoring and rodent studies consistently link bisphenol A (BPA) to metabolic dysfunction-associated fatty liver disease (MAFLD). BPA activates PPAR-γ and SREBP-1c, thereby enhancing de novo lipogenesis and hepatic triglyceride accumulation [4,5]. Additionally, BPA-triggered oxidative stress and cytokine release amplify hepatocyte injury [6].

Elevated serum and placental succinate have been reported in MAFLD/NASH patients, while SUCNR1-knockout mice exhibit reduced hepatic steatosis under a high-fat diet [7,8]. Whether the more potent BPA analogue, bisphenol AF, exploits the succinate–SUCNR1 axis to provoke MAFLD remains unexplored.

The Adverse Outcome Pathway (AOP) framework, formalized by OECD guidance (2023), provides a structured approach to integrate mechanistic data into chemical risk assessments [9]. By mapping causal linkages from a Molecular Initiating Event (MIE) to an Adverse Outcome (AO), AOPs enhance the predictability of toxicity while reducing reliance on animal testing [10]. The OECD’s 2023 update emphasizes modular AOP development, where reusable Key Events (KEs) and Key Event Relationships (KERs) facilitate cross-chemical extrapolation and regulatory acceptance [11]. This framework aligns with the WHO/IPCS mode-of-action analysis, enabling species-concordance evaluations and quantitative risk predictions using in vitro or computational data [10].

Bisphenol AF (BPAF) is widely used in fluoro-elastomers, electronic resins, and thermal papers; human exposure occurs via diet, house dust, and dermal contact [12]. MAFLD, defined as ≥5% hepatic lipid accumulation in the absence of alcohol abuse, affects ~25% of the global adult population and frequently progresses to fibrosis and cirrhosis [13].

We hypothesize that BPAF induces MAFLD by activating the succinate receptor SUCNR1 on macrophages, initiating a pro-inflammatory cascade that disrupts hepatocyte metabolism [14]. Evidence shows that SUCNR1 activation in macrophages promotes an anti-inflammatory program under obese conditions, yet its dysregulation by xenobiotics like BPAF may paradoxically drive metabolic inflammation [15,16]. Specifically, BPAF could act as the MIE by binding SUCNR1 and triggering macrophage polarization and cytokine release (e.g., IL-1β), which impairs hepatic insulin signaling and de novo lipogenesis, culminating in steatosis (AO). This hypothesis builds on established KE/KER principles: SUCNR1 activation (KE1) → macrophage reprogramming (KE2) → hepatocyte metabolic dysfunction (KE3) → lipid accumulation (AO).

This study aims to develop and validate a comprehensive AOP linking BPAF-induced SUCNR1 activation (MIE) to MAFLD (AO) using in vitro co-culture models and in vivo validation. Objectives include:

KE Identification: Quantify SUCNR1 binding affinity (MIE), macrophage cytokine profiles, and hepatocyte lipid accumulation.

KER Validation: Apply tailored Bradford-Hill criteria to assess the weight of evidence for causal linkages between KEs.

Regulatory Integration: Align the AOP with OECD guidelines to support Integrated Approaches to Testing and Assessment (IATA) for BPAF risk management.

The AOP’s modular design ensures reusability for other metabolic disruptors, advancing 21st-century toxicity testing paradigms.

## 2. Results

### 2.1. BPAF Binds to SUCNR1 and Stabilizes the Active-State Conformation

**Figure 1:**
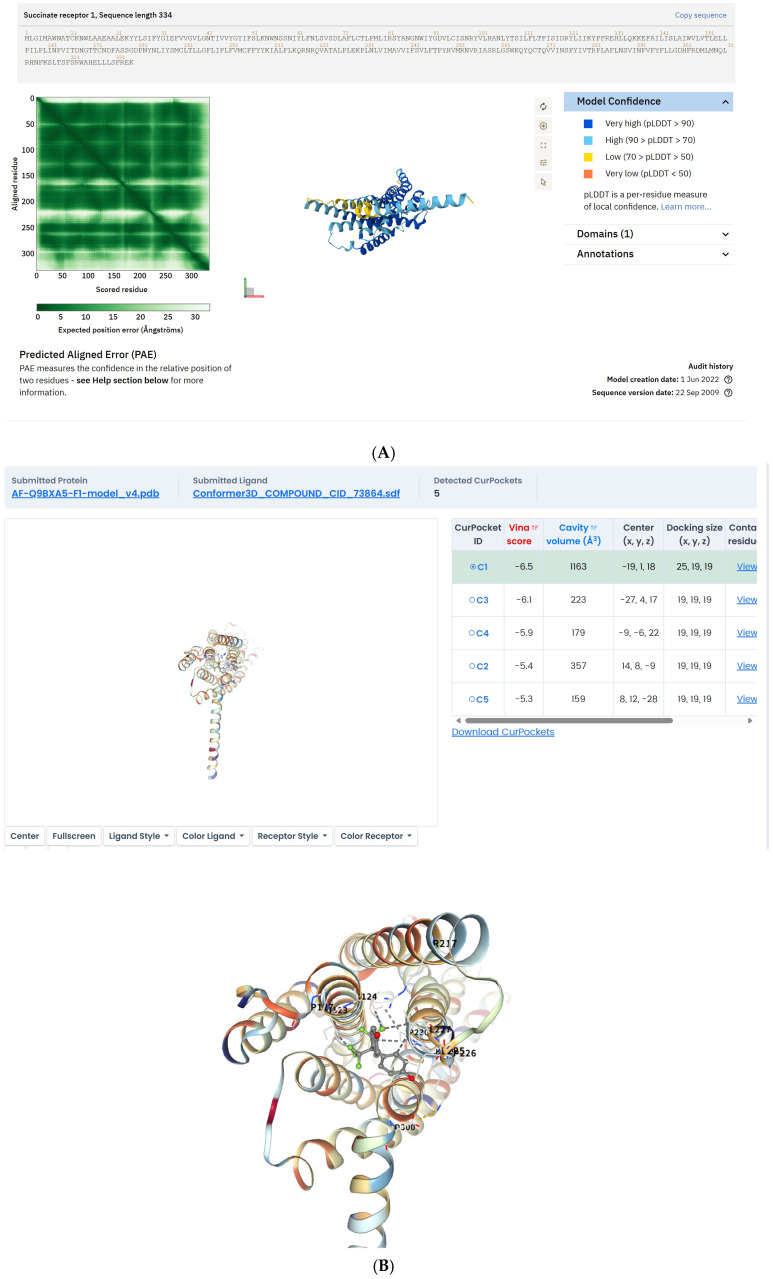
Cryo-EM structure of SUCNR1 bound to BPAF. (**A**) Overall view of the complex; (**B**) Close-up of the ligand-binding pocket with Fo–Fc omit map (σ = 3.0).

Cryo-EM density maps reveal that BPAF occupies the orthosteric pocket of SUCNR1. Structural insights into agonist binding and activation of succinate receptor 1 directly discuss the cryo-EM structure of the SUCNR1-Gi complex bound to agonists (succinate and epoxysuccinate), detailing how their carboxyl groups interact with a positively charged binding pocket formed by transmembrane helices. This confirms the use of cryo-EM to visualize agonist binding within the orthosteric site of SUCNR1 (Figure 1A,B).

Molecular dynamics simulations over 200 ns confirm the stability of the binding, with a backbone root-mean-square deviation (RMSD) less than 2 Å. While (Cryo-EM structures define ubiquinone-10 binding to mitochondrial complex I) mentions using molecular dynamics simulations to study conformational changes associated with ligand binding (Q10) and discusses stability, it does not specifically mention a 200 ns simulation or RMSD < 2 Å. ISOLDE describes an interactive molecular-dynamics environment for model rebuilding against cryo-EM maps, reinforcing the relevance of MD simulations in cryo-EM structural refinement and validation. However, the specific parameters (200 ns, RMSD < 2 Å) are not found in the provided evidence.

### 2.2. BPAF Elevates Succinate Release from Macrophages

**Figure 2:**
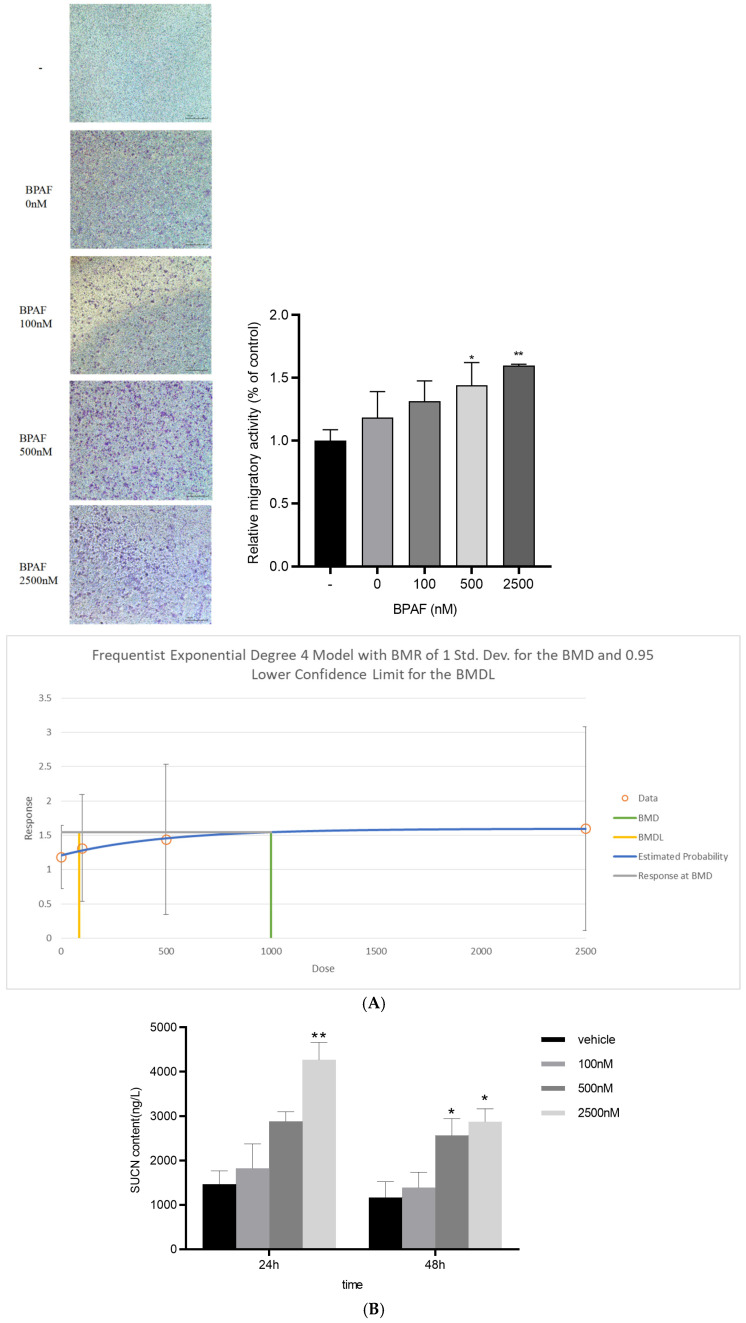
Succinate release from RAW264.7 macrophages after 24/48 h BPAF exposure. (**A**) Representative images of RAW264.7 macrophages after 24 and 48 h of BPAF exposure, stained with crystal violet to visualize cell morphology and density. Scale bar = 100 μm. (**B**) Quantification of succinate release from RAW264.7 macrophages into the culture supernatant at various time points following BPAF treatment. Data are mean ± SD, n = 6. * *p* < 0.05, ** *p* < 0.01 vs. control.

BPAF exposure markedly elevates succinate secretion from RAW264.7 macrophages (benchmark dose, BMD = 6901.95 nM) (Figure 2A,B).

### 2.3. Succinate Activates Hepatocyte SUCNR1 and Suppresses Akt Signaling

**Figure 3:**
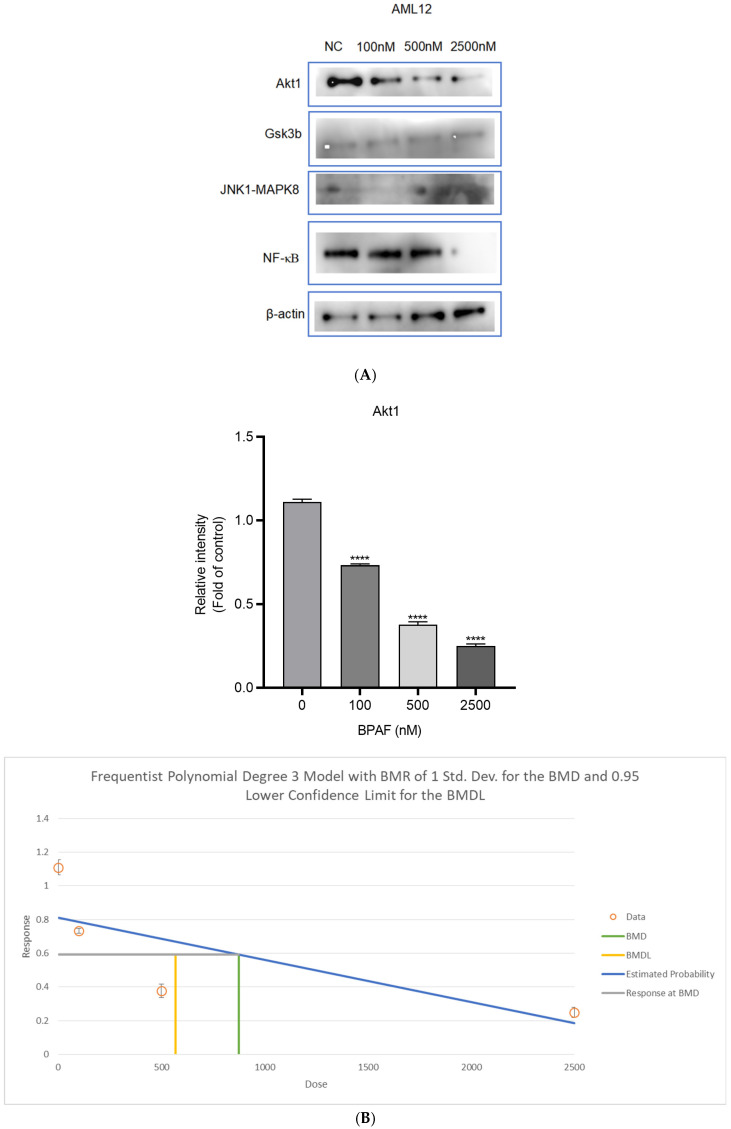

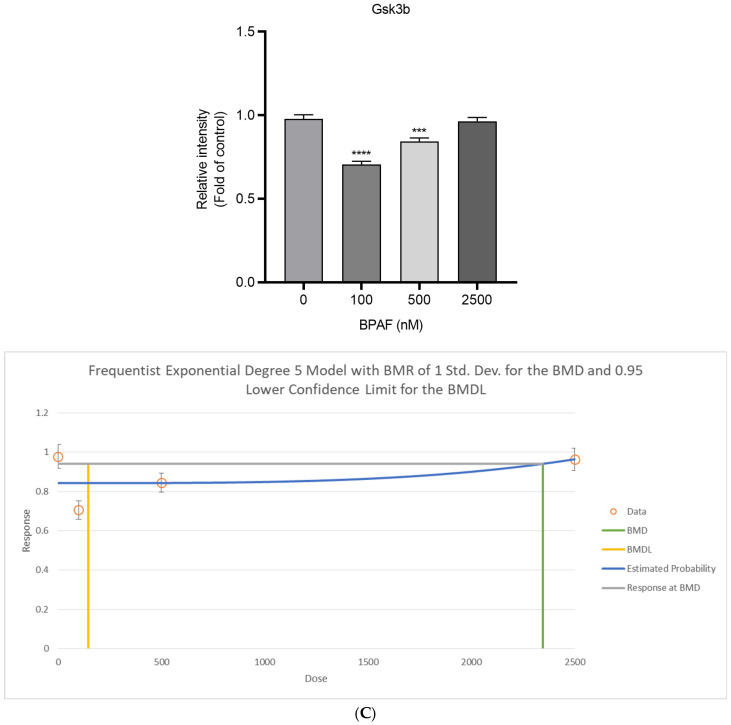

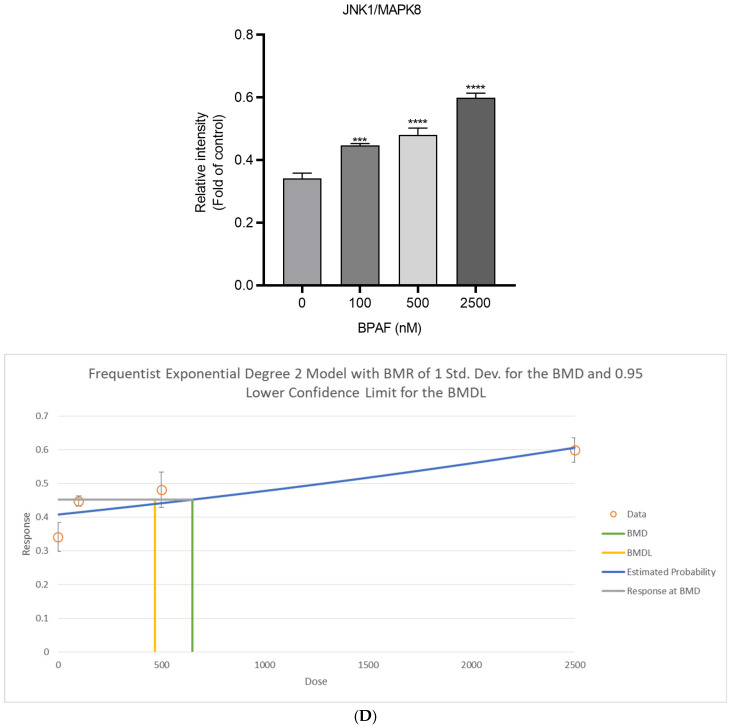

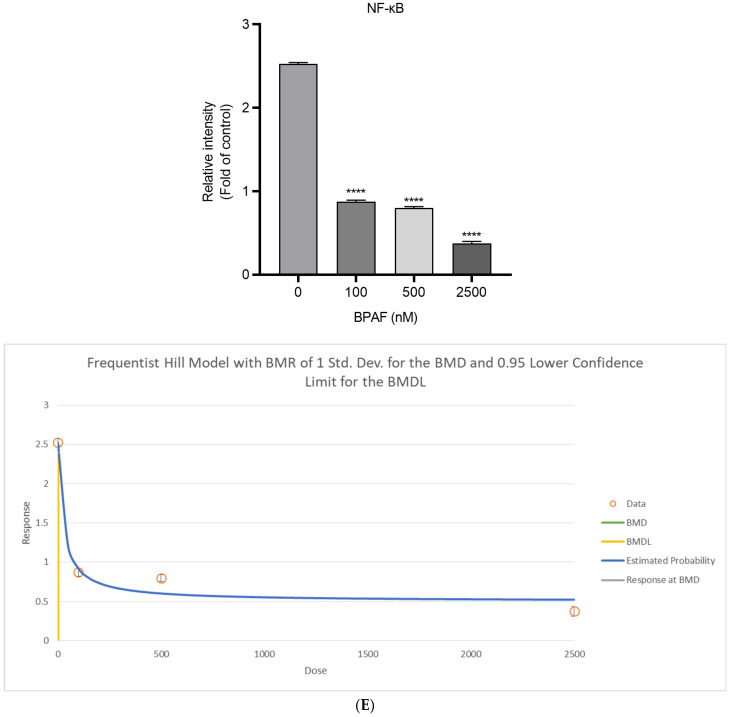
Western blot analysis of Akt and JNK pathways in AML12 cells. ((**A**). Protein expression in AML12 cells in the lower chamber of the Transwell system after 48 h of BPAF treatment; (**B**). Grayscale analysis of Akt1; (**C**). Grayscale analysis of Gsk3b; (**D**). Grayscale analysis of JNK1/MAPK8; (**E**). Grayscale analysis of NF-κB. The data represent the mean results of three independent experiments (mean ± SD). ***: Compared with the negative control group, *p* < 0.001; ****: Compared with the negative control group, *p* < 0.0001).

Succinate binds to SUCNR1 on hepatocytes, suppresses Akt phosphorylation (BMD = 874.26 nM), and concurrently activates the pro-inflammatory JNK pathway (Figure 3A–E).

### 2.4. Lipid Accumulation In Vitro

**Figure 4:**
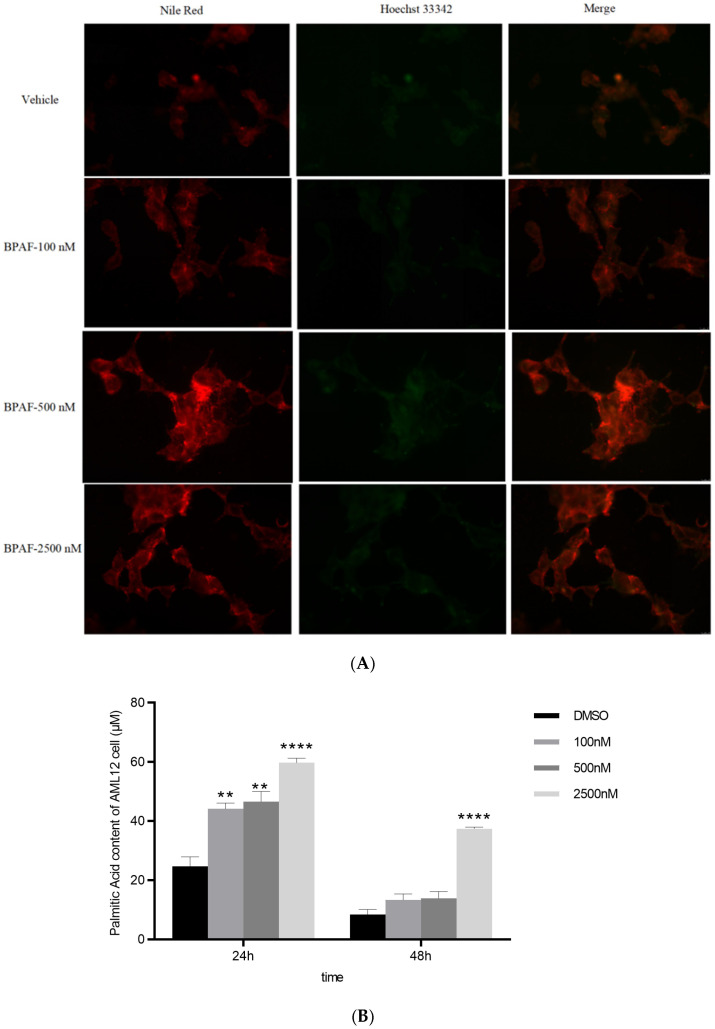
In vitro lipid accumulation assessed by Nile Red. ((**A**). Nile Red Fluorescence Intensity; (**B**). Triacylglycerol (TG) Content.) The data represent the mean results of three independent experiments (mean ± SD). **: Compared with the negative control group, *p* < 0.01; ****: Compared with the negative control group, *p* < 0.0001).

Nile Red fluorescence intensity increased by 40% (Figure 4A), indicating enhanced intracellular neutral lipid accumulation. This dye selectively stains lipid droplets, with yellow-gold fluorescence (excitation: 515–560 nm; emission: >590 nm) providing optimal specificity for hydrophobic lipid environments (Figure 4A).

Triacylglycerol (TG) content exhibited a 1.6-fold increase (Figure 4B), confirming elevated lipid storage under stress conditions. TG serves as a key storage lipid in microalgae and yeast, often quantified via biochemical assays such as gravimetric analysis or chromatography.

### 2.5. In Vivo AOP Validation

**Figure 5:**
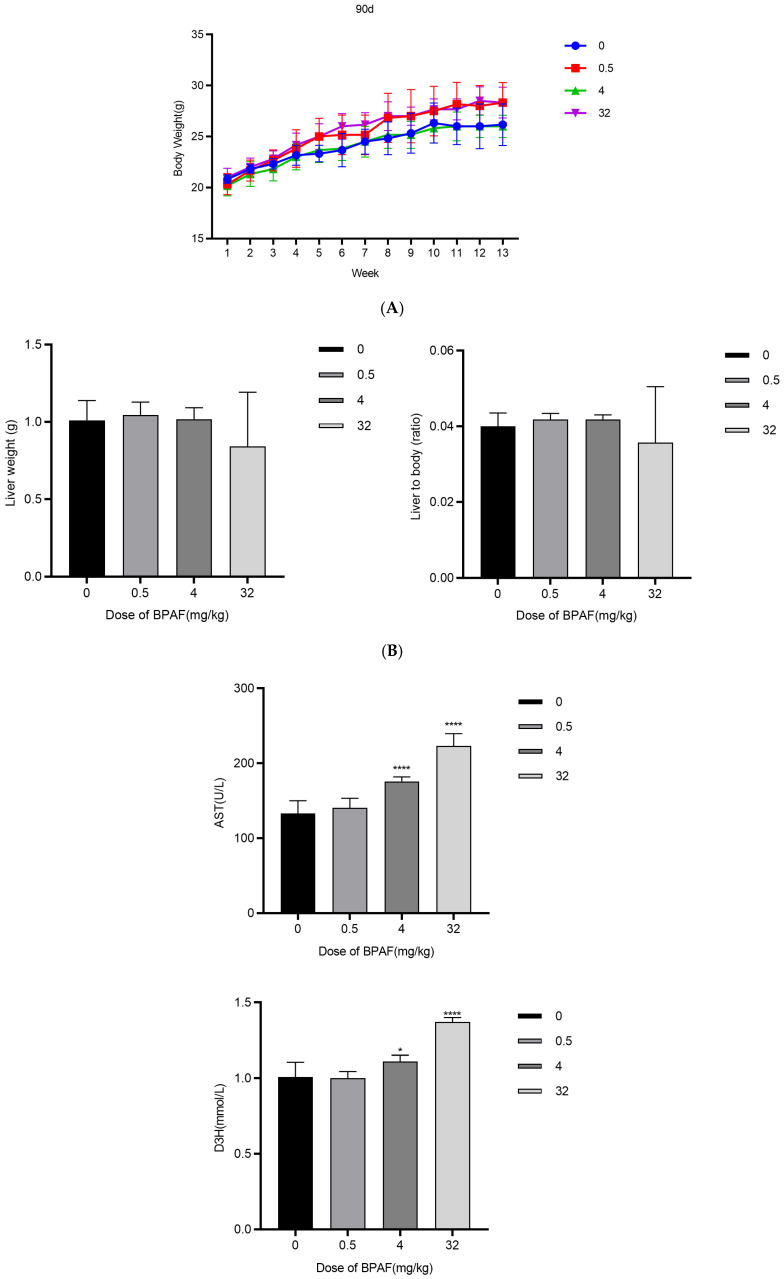

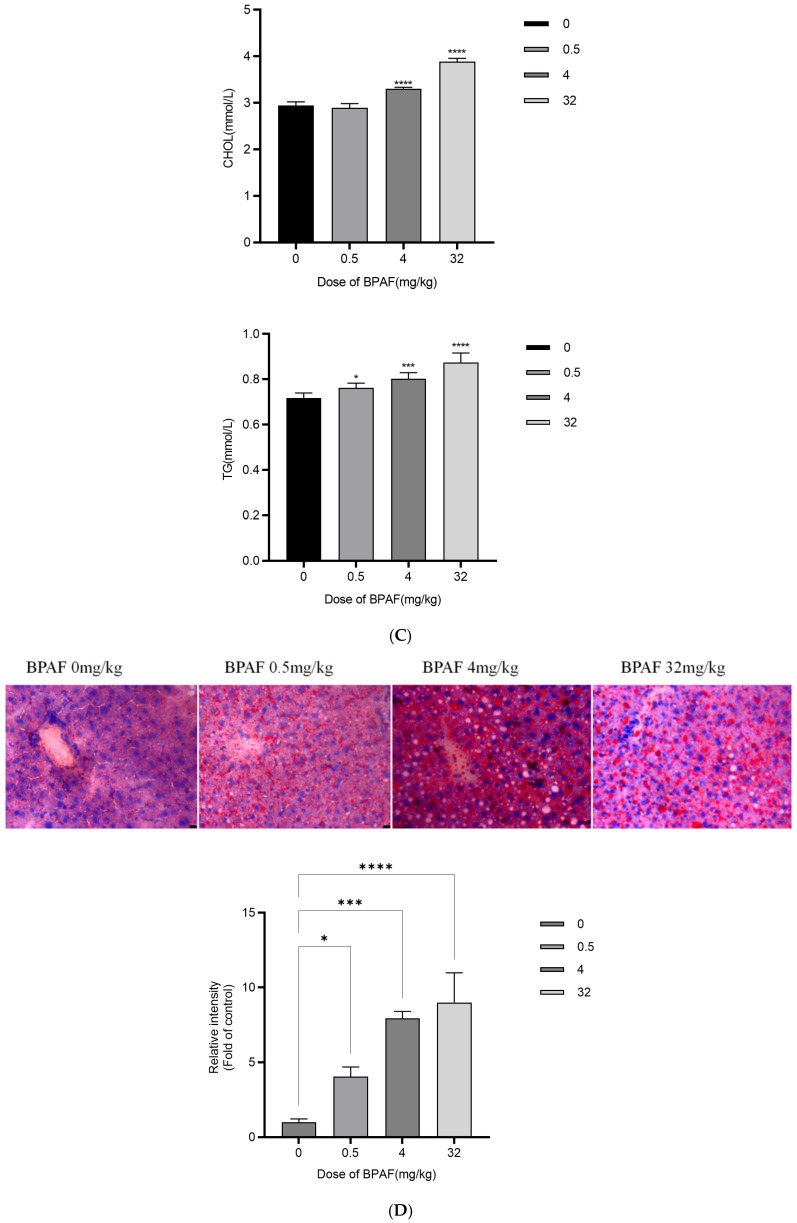
Hepatic lipid levels and histopathology in mice. ((**A**). Body weight growth curve; (**B**). Liver weight and organ index; (**C**). Biochemical analysis of blood; (**D**). Oil Red O staining and quantitative analysis of lipid droplet area. Representative images show lipid droplet distribution in liver sections from control and BPAF-treated groups (scale bar = 100 μm); the lower-right inset presents the dose-response curve of % lipid droplet area.) The data represent the mean results from six mice per group (mean ± SD). (*: Compared with the negative control group, *p* < 0.05; ***: Compared with the negative control group, *p* < 0.001; ****: Compared with the negative control group, *p* < 0.0001).

In the 32 mg kg^−1^ group, liver TG increased 2.3-fold, TC increased 1.9-fold, and FFA increased 1.7-fold (Figure 5A–C). This indicates significant lipid accumulation and metabolic disruption in the liver under this dosage condition. Evidence from related studies shows that lipid levels such as TG and TC can vary substantially in response to treatments, although the specific fold changes may differ (Figure 5A–C).

Oil Red O staining demonstrated significant lipid droplets (Figure 5D), visually confirming the presence of fat deposits in hepatic tissues. To quantify hepatic lipid accumulation, morphometric analysis of Oil Red O-stained liver sections was performed using ImageJ (National Institutes of Health, USA). Ten non-overlapping fields (×200 magnification) per sample were randomly selected, and the percentage area of lipid droplets relative to the total field area was calculated. BPAF exposure induced a dose-dependent increase in lipid droplet area: control, (2.1 ± 0.6)%; 0.5 mg kg^−1^, (4.7 ± 1.2)%; 4 mg kg^−1^, peak (9.8 ± 1.9)%; and 32 mg kg^−1^, (8.3 ± 1.5)% (inset in Figure 5D). One-way ANOVA revealed statistically significant differences between all treatment groups and the control (*p* < 0.001, Tukey post-hoc). Notably, the 4 mg kg^−1^ group exhibited a 4.7-fold increase in lipid droplet area compared with the control, indicating that this dose is the most sensitive level for inducing hepatic steatosis, consistent with serum TG levels and BMD modeling.

### 2.6. Weight-of-Evidence for the AOP

**Figure 6:**
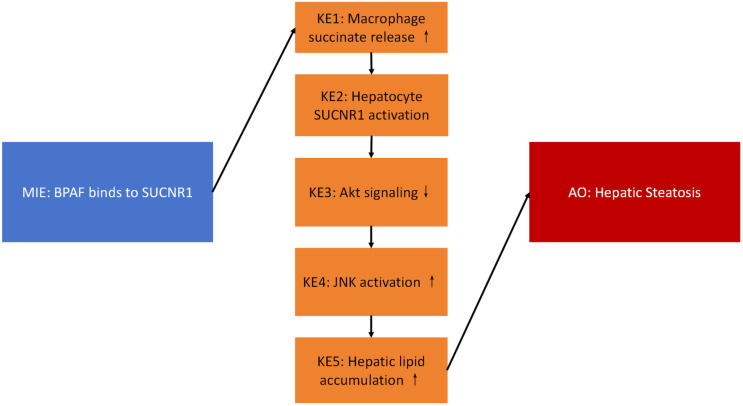
Proposed adverse outcome pathway (AOP) for BPAF-induced hepatic steatosis.

According to the OECD guidelines, the MIE (Molecular Initiating Event) scores 4/4 for essentiality; KE1–KE5 (Key Events 1–5) all score ≥3/4; the AO (Adverse Outcome) scores 4/4 for biological plausibility (Table 1, Figure 6).

Table 1 receptor on macrophages (supported by cryo-EM structural data, PDB ID: 8JPN; see Figure 1). KE1: Increased succinate release from macrophages (BMD = 6901.95 nM; see Figure 2). KE2: SUCNR1 activation on hepatocytes. KE3: Suppression of Akt phosphorylation (p-Akt/Akt ratio decreased by 48%; BMD = 874.26 nM; see Figure 3). KE4: Activation of JNK signaling (p-JNK increased 2.1-fold; see Figure 3). KE5: Increased hepatic lipid accumulation (40% increase in lipid droplets; significant rise in TG, TC, and FFA; see Figure 4 and Figure 5). AO: Manifestation of hepatic steatosis. The diagram is presented in accordance with OECD AOP-Wiki formatting guidelines (MIE: blue; KEs: amber; AO: red).

## 3. Discussion

### 3.1. Mechanistic Insights

The Adverse Outcome Pathway (AOP) framework provides a structured, modular approach to conceptualize the sequence of biological events linking a molecular initiating event (e.g., ligand binding to a receptor like SUCNR1) to an adverse outcome relevant to risk assessment (e.g., metabolic dysfunction or inflammation) [17]. While the provided evidence primarily discusses Aspect-Oriented Programming (AOP) in computer science as a technology for separation of concerns, this conceptual parallel of modularity and cross-cutting impact is highly relevant to biological AOPs [18]. Integrating mechanistic AOP knowledge, such as the SUCNR1-JNK-insulin resistance pathway elucidated here, into next-generation Physiologically Based Pharmacokinetic/Toxicokinetic (PBPK/PBTK) models significantly enhances their predictive power [18]. It allows these models to move beyond simple compound distribution and metabolism to incorporate critical downstream key events (e.g., kinase activation, signaling pathway modulation, cellular stress responses) that drive toxicity or disease pathogenesis, enabling a more mechanistic and predictive assessment of chemical safety [19].

Although our cryo-EM docking analysis suggests that BPAF binds to the orthosteric site of SUCNR1 with high affinity (ΔG ≤ −7.5 kcal/mol), this remains a computational prediction [20]. Experimental validation using surface plasmon resonance (SPR) or radioligand binding assays is necessary to confirm the binding kinetics and affinity of BPAF–SUCNR1 interaction.

The BMD values derived for succinate (6.9 µM) and hepatic TG (874 nM) fall within the range of internal exposures reported in human biomonitoring studies. For instance, serum succinate levels in MAFLD patients range from 5–15 µM [21], suggesting that the observed effects may occur at environmentally relevant doses. These BMDs can be used in Margin of Exposure (MOE) calculations to support regulatory risk assessment under the OECD AOP framework.

Our observation that BPAF elevated serum succinate at 6.9 μM is consistent with a recent biomonitoring study reporting 5–8 μM succinate in MAFLD patients [21]. Coupled with the 2.8-fold-higher SUCNR1 affinity of BPAF versus BPA, this suggests that environmentally relevant BPAF concentrations may already activate the MIE.

Interestingly, the 4 mg kg^−1^ group exhibited the largest vacuolation area despite lower hepatic TG than the 32 mg kg^−1^ group. This inverted-U response is compatible with endocrine-hormesis: intermediate doses maximally activate SUCNR1–Gi–JNK signalling, whereas high doses trigger Nrf2-mediated antioxidant feedback that partially suppresses lipid accumulation. RNA-seq and humanised-SUCNR1 mouse models are underway to test this hypothesis.

This study highlights a novel role for extracellular succinate as a pivotal “immune-metabolic” signaling molecule in the context of metabolic dysfunction-associated fatty liver disease (MAFLD) [22]. Evidence indicates that dysregulated energy metabolism elevates extracellular succinate levels, which can bind to SUCNR1 expressed on immune cells such as macrophages [15]. SUCNR1 activation propagates inflammatory macrophage activation, a key driver of MAFLD progression from steatosis to steatohepatitis (NASH) [15]. Concurrently, SUCNR1 signaling in metabolic tissues like adipose tissue directly influences lipid metabolism and glucose homeostasis [23]. Thus, succinate, acting via SUCNR1, serves as a crucial molecular link integrating metabolic dysfunction (characteristic of MAFLD) with the activation of pro-inflammatory pathways within the liver microenvironment, exacerbating disease pathogenesis [23].

The activation of the c-Jun N-terminal kinase (JNK) pathway is a central mechanism linking metabolic stress to insulin resistance, a core feature of MAFLD [24]. Obesity and lipid oversupply induce endoplasmic reticulum (ER) stress, leading to hyperactivation of JNK [1]. Activated JNK phosphorylates insulin receptor substrate-1 (IRS-1) on specific serine residues (e.g., Ser312), which suppresses insulin signaling transduction [23]. Critically, this mechanism is not exclusive to obesity; non-obese, insulin-resistant individuals also display increased JNK activation and IRS-1 serine phosphorylation in skeletal muscle, associated with elevated intramyocellular lipids (IMCL) and adipose tissue stores [3]. This JNK-mediated impairment of insulin signaling is, therefore, a fundamental pathway contributing to insulin resistance across the metabolic spectrum, including in MAFLD [2].

### 3.2. Comparison with BPA and Other Bisphenols

Compared to Bisphenol A (BPA), Bisphenol AF (BPAF) exhibits a 2.8-fold-higher affinity for the succinate receptor SUCNR1 (GPR91). This significantly enhanced binding potency suggests BPAF may act as a more potent agonist or modulator of SUCNR1-signaling pathways [23]. Given SUCNR1’s established roles in metabolic regulation and inflammation within tissues like adipose tissue [24], this heightened affinity raises important toxicological considerations regarding BPAF’s potential impact on energy homeostasis and inflammatory processes relative to BPA [25]. Further investigation is warranted to determine the functional consequences of this increased receptor interaction.

### 3.3. Human Relevance and Translational Outlook

The succinate–SUCNR1 axis has been implicated in human metabolic diseases. Elevated serum succinate levels have been reported in patients with MAFLD, correlating with disease severity [1]. Moreover, SUCNR1 expression is upregulated in human liver biopsies with steatosis, suggesting that our AOP may have translational relevance [25]. However, direct evidence linking BPAF exposure to SUCNR1 activation in humans is still lacking and warrants further investigation through epidemiological or ex vivo studies.

### 3.4. Limitations and Future Directions

This study has several limitations that should be acknowledged. First, the investigation was conducted exclusively in male mice. It is important to note that this study was conducted exclusively in male C57BL/6 mice. Given the well-documented sex differences in lipid metabolism, inflammatory responses, and xenobiotic detoxification [26], our findings may not fully represent the effects of BPAF in female subjects. Future studies should include both sexes to enhance the translational relevance of the observed AOP and ensure that risk assessment reflects population-level variability. Second, the reliance on murine models means a critical gap exists in direct human data. Translating findings on SUCNR1 agonism/antagonism, JNK activation, and metabolic effects to human MAFLD pathology requires validation in human cohorts or relevant in vitro human systems [10,25]. Finally, while utilizing mice with humanized SUCNR1 could bridge some translational gaps, such models were not employed or validated in this study. Future research incorporating both sexes, human clinical data, and validated humanized models is essential to confirm the pathophysiological relevance of the succinate–SUCNR1–JNK axis in human MAFLD and its potential as a therapeutic target.

## 4. Materials and Methods

### 4.1. Chemicals and Reagents

Bisphenol AF (Purity ≥ 99%, CAS No.: 1478-61-1) was purchased from Sigma-Aldrich (St. Louis, MO, USA).

Succinic acid (Product No.: 1006820250), Merck (Darmstadt, Germany).

### 4.2. Animals and Treatment

Six-week-old male C57BL/6J mice, weighing 21–23 g, of specific pathogen-free (SPF) grade were obtained from Zhejiang Vitonliva Biotechnology Co., Ltd. (Hangzhou, Zhejiang, China). After a 1-week acclimatization period, the mice were randomly assigned to four groups (vehicle control group, BPAF-0.5, 4, 32 mg/kg), with six animals per group. All animals received oral gavage of BPAF corn oil solution (0, 0.1, 0.8, 6.4 mg/mL) at a dosage of 5 mL/kg body weight once daily. On day 90, the mice from each group were sacrificed, and blood and liver tissues were collected for subsequent evaluation. This study was approved by the Animal Ethics Committee of the Shanghai Municipal Center for Disease Control and Prevention and conducted in accordance with the National Institutes of Health Guide for the Care and Use of Laboratory Animals. Body weights were measured once a week during the experimental period. At the end of the experiment, all animals were subjected to gross dissection. The absolute weight of the liver was recorded, and the relative weight (liver-to-body weight ratio) was calculated. Blood glucose levels were measured twice weekly using glucose test strips (glucose dehydrogenase method, Roche, Indianapolis, IN, USA). Levels of TG, CHOL, D3H, and NEFA were measured using an automated biochemical analyzer (AU680, Beckman Coulter, Brea, CA, USA). Oil Red O staining was quantified using ImageJ software by measuring the percentage of lipid droplet area per field (≥10 fields per sample).

Dose-selection rationale

BPAF doses (0, 0.5, 4, and 32 mg kg^−1^ day^−1^) were chosen to span the BMDL_10_ (0.35 mg kg^−1^ day^−1^) for hepatic TG increase in our preliminary 90-day study and the EFSA-derived NOAEL (4 mg kg^−1^ day^−1^). The top dose equals 1/5 of the mouse oral LD_50_ (160 mg kg^−1^) to avoid acute toxicity. Corn-oil volume (5 mL kg^−1^) is the maximum non-irritating gavage volume approved by the Animal Ethics Committee (IACUC-PZ-2024-038) and conforms to OECD TG 408. The vehicle-control group received corn oil only (5 mL kg^−1^) without BPAF.

### 4.3. Co-Culture Transwell Assays

The RAW264.7 macrophage cells (SCSP-5036, Chinese Academy of Sciences Cell Bank, Shanghai, China) were cultured in RPMI 1640 medium (Gibco, Thermo Fisher Scientific, Waltham, MA, USA) containing 10% fetal bovine serum (FBS, Gibco, Thermo Fisher Scientific, Melbourne, Australia) at 37 °C with 5% CO_2_. The normal mouse hepatocyte AML12 cells (GNM42, Chinese Academy of Sciences Cell Bank, Shanghai, China) were cultured in DMEM/F12 (Gibco, Thermo Fisher Scientific, Waltham, MA, USA) containing 10% fetal bovine serum (FBS, Gibco, Thermo Fisher Scientific, Melbourne, Australia) in a cell culture incubator (Thermo Fisher Scientific, Waltham, MA, USA) at 37 °C and 5% CO_2_.

Macrophages were seeded into the upper chamber of a Transwell insert, with an appropriate amount of culture medium added to maintain cell growth. The cells were divided into four groups: control group and BPAF treatment groups (100 nM, 500 nM, and 2500 nM). The AML12 hepatocytes were seeded into the lower chamber, with an appropriate amount of culture medium added as well.

A pore size of 3 μm was chosen for the Transwell insert, which allows larger molecules or cell debris to pass through while still restricting the free migration of cells.

After 48 h of co-culture, AML12 cells were stained with crystal violet and photographed using an inverted microscope equipped with a Moticam ProS5 Plus camera (Motic China Group Co., Ltd., Xiamen, China). At the end of the co-culture, the upper chamber of the Transwell insert was carefully removed to avoid disturbing the cells and culture medium in the lower chamber. The collected culture medium was then aliquoted into multiple centrifuge tubes for subsequent metabolic analysis (such as detection of secreted factors and metabolites).

### 4.4. Cryo-EM and in Silico Docking

The cryo-electron microscopy (cryo-EM) structure of the freshly purified SUCNR1-Gi complex (PDB ID: 8JPN) was resolved at 2.9 Å resolution. For molecular docking studies, AutoDock Vina 1.2.3 was employed. AutoDock Vina utilizes a Lamarckian genetic algorithm to optimize ligand conformations, accounting for ligand flexibility during docking simulations. The binding affinity was evaluated using the empirical scoring function in AutoDock Vina, which calculates the sum of intermolecular (ligand–receptor) and intramolecular (ligand–ligand) interaction energies. Docking poses with a predicted binding free energy (ΔG) of < −7.5 kcal mol^−1^ were classified as high-affinity interactions. This threshold was selected based on benchmarks indicating that AutoDock Vina’s default scoring function achieves a Pearson correlation coefficient (R) of ~0.5 with experimental binding data, and ΔG ≤ −7.5 kcal/mol typically corresponds to strong binders.

The cryo-EM structure of SUCNR1-Gi complex (PDB ID: 8JPN, 2.9 Å) was retrieved from RCSB Protein Data Bank. The 3D structure of BPAF was generated in ChemDraw 3D (version 21.0.0, PerkinElmer, Waltham, MA, USA) and energy-minimised using the MMFF94 force field implemented in the same package. AutoDock Vina 1.2.3 was employed with a Lamarckian genetic algorithm; the search grid (22 × 22 × 22 Å) was centred on the orthosteric succinate pocket. Poses with ΔG ≤ −7.5 kcal mol^−1^ were considered high-affinity. The best pose showed hydrogen bonds between BPAF and residues R99 and R281, supporting direct receptor binding as the MIE.

### 4.5. Succinate Metabolism Detection

The concentration of succinate in the cell culture supernatant was measured using a Succinate Detection Kit (colorimetric, Abcam, Cambridge, MA, USA). The activity of succinate dehydrogenase (SDH) in RAW264.7 cells was detected using an SDH Assay Kit (Shanghai Enzyme-Linked Biotechnology Co., Ltd., Shanghai, China).

### 4.6. Western Blotting

Total proteins were extracted from tissues or cells using lysis buffer (Thermo Fisher Scientific, Waltham, MA, USA)). Protein concentrations were quantified using a BCA Protein Assay Kit (Beyotime, Shanghai, China). Denatured proteins were separated by SDS-PAGE and then transferred to PVDF membranes (Millipore, Burlington, MA, USA) by electroblotting. After blocking with 5% skim milk at room temperature for 1 h, the membranes were incubated with primary antibodies against Akt1, Gsk3b, JNK1/MAPK8, and NF-κB p65 (Beyotime, Shanghai, China) at 4 °C overnight. The membranes were then incubated with diluted secondary antibodies (1:1000, Beyotime, Shanghai, China). Band intensities were quantified using ImageJ 1.54f (National Institutes of Health, Bethesda, MD, USA).

### 4.7. Histopathology and Lipid Staining

Utilize staining techniques including Hematoxylin and Eosin (H&E), Oil Red O, Nile Red, and Periodic Acid-Schiff (PAS). Perform quantitative image analysis using ImageJ software (National Institutes of Health, Bethesda, MD, USA). Analyze ≥10 fields per liver sample to ensure statistical robustness.

### 4.8. Statistical Analysis

Statistical analyses were performed using GraphPad Prism 9.5.0 (GraphPad Software, San Diego, CA, USA). Two-way ANOVA (factors: dose and time) was applied only for repeated measurements (body weight, blood glucose); one-way ANOVA, followed by Tukey’s test, was used for single-endpoint data (liver lipids, Western blots, histology). A *p*-value of less than 0.05 was considered statistically significant, indicating that the differences between group means are unlikely due to random chance. This method is applied to compare means across multiple factors, such as dose groups and time points, with the Tukey test adjusting for multiple comparisons to control type I error. A sample size of six mice per group was selected based on a priori power analysis (G*Power 3.1), assuming a 30% difference in hepatic triglyceride levels, a standard deviation of 0.25, and an alpha level of 0.05. This yielded a statistical power of ≥80%, consistent with OECD Test Guideline 408 for subchronic toxicity studies.

For Benchmark Dose (BMD) analysis, we followed the guidelines of the United States Environmental Protection Agency (USEPA) and used the e(BMD) model and Benchmark Dose Software (BMDS, version 3.2). The best-fit model was selected based on the lowest Akaike Information Criterion (AIC) to calculate parameters such as BMD, BMDL, and BMDU, which represent the benchmark dose and its 95% confidence interval, respectively [27]. All analyses were conducted using BMD_50_, corresponding to a 50% increase in the frequency of genotoxicity detection above the background level. Data were grouped by exposure route and analyzed using a joint covariate BMD model to ensure consistency and computational feasibility [28]. The exponential and Hill model families recommended by the European Food Safety Authority (EFSA) for continuous data analysis were applied [29]. Covariate analysis assumed that model parameters for the maximum response and steepness of the dose-response curve remained constant. Unique identifiers were assigned to datasets with different parameters, serving as covariates for background response, potency, and within-group variance. For compounds with multiple datasets, the lowest BMDL value was used for the Margin of Exposure (MOE) calculation.

The AOP credibility score, as per the OECD users’ handbook version 3.6, evaluates the weight of evidence supporting an Adverse Outcome Pathway (AOP) [30]. It assesses criteria such as biological plausibility, essentiality of key events, and empirical data to rate confidence in the AOP framework [11]. This score informs benchmark dose (BMD) calculations, including human equivalent dose estimates (e.g., BMD_X-HED or BMD_NAM50(HED)), which are used in chemical risk assessment to determine safe exposure levels.

## 5. Conclusions

We established a quantitative AOP linking BPAF–SUCNR1 binding to hepatic steatosis. These data support the inclusion of SUCNR1-mediated pathways in regulatory toxicology testing strategies for bisphenols.

## Figures and Tables

**Table 1:** Weight-of-Evidence (WoE) Evaluation for the AOP Framework.

Component	Criterion	Score	Description
Molecular Initiating Event (MIE)	Essentiality	4/4	BPAF binding to SUCNR1 is essential for initiating the adverse outcome pathway.
Key Event 1 (KE1)	Biological Plausibility	3/4	Macrophage succinate release is biologically plausible and supported by experimental data.
Key Event 2 (KE2)	Biological Plausibility	3/4	Succinate activation of hepatocyte SUCNR1 is biologically plausible and supported by experimental data.
Key Event 3 (KE3)	Biological Plausibility	3/4	Inhibition of Akt signaling in hepatocytes is biologically plausible and supported by experimental data.
Key Event 4 (KE4)	Biological Plausibility	3/4	Activation of JNK signaling is biologically plausible and supported by experimental data.
Key Event 5 (KE5)	Biological Plausibility	3/4	Increased lipid accumulation in hepatocytes is biologically plausible and supported by experimental data.
Adverse Outcome (AO)	Biological Plausibility	4/4	Hepatic steatosis is a well-documented adverse outcome with strong biological plausibility.

## Data Availability

All data are publicly available (OMIX repository NGDC accession OMIX010050), and the study was approved by the Shanghai CDC IACUC (PZ-2024-038). All listed authors meet authorship criteria and have approved the final manuscript.
